# Controlled Size Characterization Process for In-Situ TiB_2_ Particles from Al Matrix Composites Using Nanoparticle Size Analysis

**DOI:** 10.3390/ma17092052

**Published:** 2024-04-27

**Authors:** Mingliang Wang, Qian Wang, Zeyu Bian, Siyi Chen, Yue Gong, Cunjuan Xia, Dong Chen, Haowei Wang

**Affiliations:** 1 State Key Laboratory of Metal Matrix Composites, Shanghai Jiao Tong University, Shanghai 200240, China; cythia-cd4@sjtu.edu.cn (S.C.); xiacunjuan@sjtu.edu.cn (C.X.); chend@sjtu.edu.cn (D.C.); hwwang@sjtu.edu.cn (H.W.); 2 Institute of Alumics Materials, Shanghai Jiao Tong University (Anhui), Huaibei 235000, China; 3 Anhui Province Industrial Generic Technology Research Center for Alumics Materials, Huaibei Normal University, Huaibei 235000, China; 4 Shanghai Institute of Satellite Engineering, No. 3666 Yuanjiang Road, Shanghai 201109, China; 5 Innovation Academy for Microsatellites of Chinese Academy of Sciences, Shanghai 201203, China; bianzeyu@microsate.com

**Keywords:** metal matrix composites, size characterization, photon correlation spectroscopy, particle dispersion, dispersion mechanisms

## Abstract

The wide size range and high tendency to agglomerate of in-situ TiB_2_ particles in reinforced Al matrix composites introduce great difficulties in their size characterization. In order to use a nanoparticle size analyzer (NSA) to obtain the precise size distribution of TiB_2_ particles, a controlled size characterization process has been explored. First, the extraction and drying processes for TiB_2_ particles were optimized. In the extraction process, alternated applications of magnetic stirring and normal ultrasound treatments were proven to accelerate the dissolution of the Al matrix in HCl solution. Furthermore, freeze-drying was found to minimize the agglomeration tendency among TiB_2_ particles, facilitating the acquisition of pure powders. Such powders were quantitatively made into an initial TiB_2_ suspension. Second, the chemical and physical dispersion technologies involved in initial TiB_2_ suspension were put into focus. Chemically, adding PEI (M.W. 10000) at a ratio of m_PEI_/m_TiB2_ = 1/30 into the initial suspension can greatly improve the degree of TiB_2_ dispersion. Physically, the optimum duration for high-energy ultrasound application to achieve TiB_2_ dispersion was 10 min. Overall, the corresponding underlying dispersion mechanisms were discussed in detail. With the combination of these chemical and physical dispersion specifications for TiB_2_ suspension, the bimodal size distribution of TiB_2_ was able to be characterized by NSA for the first time, and its number-average diameter was 111 ± 6 nm, which was reduced by 59.8% over the initial suspension. Indeed, the small-sized and large-sized peaks of the TiB_2_ particles characterized by NSA mostly match the results obtained from transmission electron microscopy and scanning electron microscopy, respectively.

## 1. Introduction

By virtue of their outstanding mechanical properties, in-situ TiB_2_ particle-reinforced Al composites offer broad applications in the fields of national defense, the military industry, aerospace, and aviation [1,2,3,4,5]. For particle-reinforced composites, a good understanding of the size distribution of the particles is significant to technical improvements in and property manipulation of the composites [6,7,8,9]. At present, the size distributions for in-situ TiB_2_ particles derived from statistical methods mainly utilize data from sources including scanning electron microscopy (SEM) [10,11,12], transmission electron microscopy (TEM) [13,14,15,16,17], X-ray diffraction analysis [14], and photon correlation spectroscopy (PCS) [18,19,20]. 

The electron microscopy methods (i.e., SEM and TEM) focus on the interaction between the electron beam and the samples, and can give direct morphological information about the particles [21,22]. The accuracy of the size results obtained from this method, however, is limited by the manual statistical errors and the field range of the electron microscope. X-ray diffraction analysis mainly relies on the broadening of X-ray diffraction peaks or the change in small-angle diffraction angles for size characterization [23,24]. Considering that the sizes of the crystal grains are assumed to be the diffraction columns parallel to the diffraction direction in X-ray diffraction analysis, the characterized size results from this method are inclined to be larger than the actual size [25]. In addition, the electron microscopy methods and X-ray diffraction analysis all have the problem of only being capable of a narrow size measurement range, which also leads to a significant difference in the size results obtained from these methods [26,27]. For example, the average diameters of TiB_2_ particles from in-situ TiB_2_/Al composites characterized by SEM, TEM, and XRD were 180 nm, 78 nm, and 40 nm, respectively [14,16]. These disadvantages of the electron microscopy methods and X-ray diffraction analysis make it difficult for them to handle the wide size range of in-situ TiB_2_ particles. 

The nanoparticle size analyzer (NSA), which is based on the PCS method and has a measurement range of 0.6–6000 nm, is expected to give accurate size information for in-situ TiB_2_ particles [28,29]. However, achieving a particle stable dispersion state in the liquid phase is the precondition to obtaining precise size information by NSA [30]. Otherwise, the particle size obtained by NSA will be larger than its actual size. Nevertheless, current studies which used NSA did not focus on the dispersion state of TiB_2_ particles in the suspension. Therefore, their size results will be impacted by TiB_2_ agglomeration [19,20,31]. For instance, Xiao et al. used both NSA and TEM methods to obtain size information for in-situ TiB_2_ particles. The size range measured by NSA was around 100–700 nm, while it was observed that most TiB_2_ particles were less than 100 nm in TEM images [18]. Generally, both results for the particles’ size were not comparable.

In order to obtain a precise size distribution of in-situ TiB_2_ particles by NSA, this work explored a controlled size characterization process, and it was concentrated on the dispersion state of the TiB_2_ suspension. The extraction and drying processes for TiB_2_ particles were optimized. The dispersion techniques and mechanisms for TiB_2_ suspension were both studied and discussed. Based on the considerable dispersion state achieved for the TiB_2_ particles, the optimized size result characterized by NSA was compared with those obtained by SEM and TEM.

## 2. Materials and Methods

**Figure 1:**
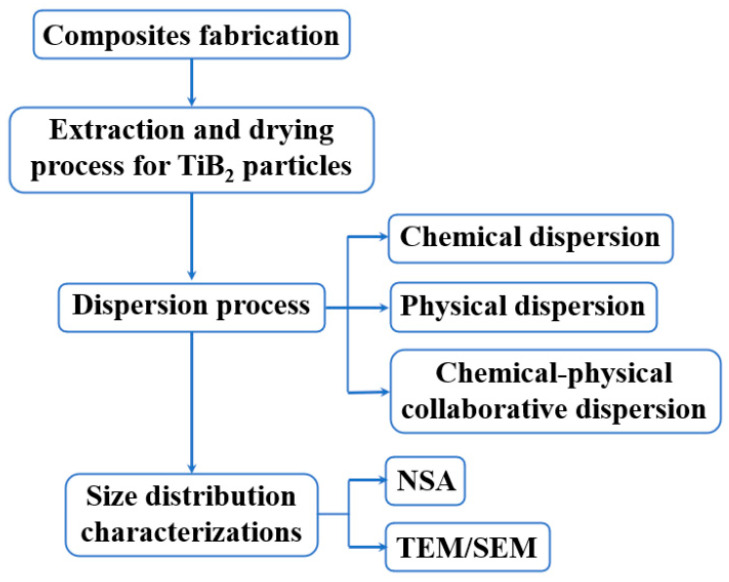
Experimental roadmap.

The main experimental process in this work to control the size characterization process for in-situ TiB_2_ particles used in Al matrix composites using the NSA method is shown in Figure 1. The specific operation details are explained in the next few sections.

### 2.1. Composite Fabrication

A 5 wt.% TiB_2_/Al composite was produced by the in-situ mixed salt route [32]. Firstly, a pre-weighted mixture of salts comprising K_2_TiF_6_ (Jiuding Fluorin chemicals, Longyan, China) and KBF_4_ (Jiuding Fluorin chemicals, China) were adding into the molten Al at 850 °C, and the melt was stirred for 30 min to synthesize TiB_2_ particles. After skimming out the slag completely, the melt was poured into a mold to obtain the as-cast TiB_2_/Al composite ingot.

### 2.2. Extraction and Drying Process for Obtaining TiB_2_ Particles

Cutting a ~5 g sample from the as-cast TiB_2_/Al ingot, the sample was completely dissolved in 20 wt.% HCl solution in a breaker. The total molar mass of HCl (Sinopharm Group, Beijing, China) is ~4 times larger than the theoretical molar mass required to completely react with the Al in the composite. Furthermore, all chemical reagents in this work were used as obtained without further purification.

Once the reaction speed was observed to slow down significantly, either magnetic stirring or normal ultrasound treatment (120 W) was applied alternatively to accelerate the reaction. When the dissolution process was completed, the acidic TiB_2_ suspension was subjected to vacuum filtration operation [33]. Critically, multiple filter membranes were overlapped to reduce the loss of particles in this operation. The filtration operation should be repeated 3–4 times to obtain the neutral condition for particle suspension. After the vacuum filtration operation, the TiB_2_ particles attached to the filter membrane were sonicated into the deionized water. According to our trial tests, there is no risk of changing the size distribution of the measured particles by losing the fine particles through this filtration technique. Finally, the obtained neutral TiB_2_ suspension was put into a freeze dryer or vacuum dryer to obtain pure TiB_2_ powder.

### 2.3. Dispersion Process of TiB_2_ Particles

A quantitative amount of pure TiB_2_ powder was added into the deionized water, using the normal ultrasound treatment (120 W), to obtain the initial TiB_2_ suspension (0.2 mg/mL). This is deemed the no-surfactant-treated suspension in its undispersed state. The subsequent chemical or physical dispersion processes are performed on this initial TiB_2_ suspension (undispersed state):

- For chemical dispersion, the surfactant solution of a certain concentration was added to the initial TiB_2_ suspension and then mixed evenly using the normal ultrasound treatment (120 W). The HCl and NaOH (Sinopharm Group, China) solutions were used to adjust the pH of the suspension.
- For physical dispersion, a high-energy ultrasound treatment (300 W) was used to explore the dispersion effects of different ultrasound durations on the initial TiB_2_ suspensions.

### 2.4. Material Characterization

The NSA (Zetasizer Nano S, Worcestershire, UK), SEM (Maia3 Model 2016, Tescan, Brno, Czech), and TEM (JEM2100, JEOL, Akishima, Japan) techniques were used for the size distribution measurement of TiB_2_ particles. For the NSA characterization, the TiB_2_ suspension (0.2 mg/mL) was tested 10 times, and the results of the largest and smallest number-average diameters were removed. Then, the remaining 8 results were averaged to obtain the final size distribution. The zeta potential of the TiB_2_ in the suspension was also measured by the NSA equipment.

For both the SEM and TEM characterizations, the following statistical principles were adopted: (1) To exclude particles which have an unobvious border or are severely agglomerated; (2) To select multiple pictures for statistics. For SEM, there were ~600 TiB_2_ particles in 5 SEM images, and ~300 TiB_2_ particles in 20 TEM images for the corresponding size distribution analyses.

## 3. Results & Discussion

### 3.1. Extraction and Drying Process of TiB_2_ Particles

#### 3.1.1. Extraction Process Optimization

Both HCl and NaOH solutions are the typical reagents used to dissolve the Al matrix in a TiB_2_/Al composite. In this work, the HCl solution was chosen as the dissolving reagent in the extraction experiment under the following considerations: (1) The reaction rate of Al and HCl is found to be greater than that of Al and NaOH at the same solution concentration; (2) It is necessary for the NaOH solution to maintain sufficient concentration during the dissolving process to avoid Al(OH)_3_ precipitation [34]; (3) NaOH has a corrosive effect on glass equipment, which is widely used for subsequent filtration process; (4) HCl can also dissolve intermediate products, such as Al_3_Ti [31].

**Figure 2:**
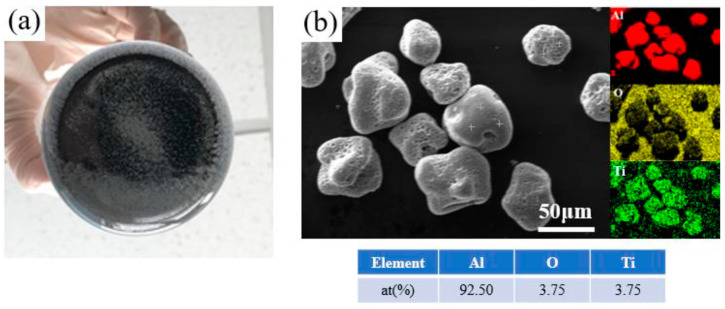
(**a**) White residual particles during the dissolution process of in-situ TiB_2_/Al composites; (**b**) SEM image with element mapping and average composition characterization of the white particles.

In the preliminary extraction experiment, after using the HCl solution to react with Al matrix for 12 h, there were still many white residual particles observed at the bottom of the beaker (Figure 2a). These white particles rarely decreased with an increase in the dissolution duration. This phenomenon indicates that the reaction rate between the white particles and HCl solution should be slow.

In order to explore the composition of the white particles, they were collected and studied. The morphological analysis shows that most of these particles are irregularly spherical and 60–80 μm in size (Figure 2b). There are many holes distributed on the particle’s surface. From the results of element mapping and average composition characterization (Figure 2b), it can be inferred that the white particles are Al particles. Furthermore, there are a few Ti elements in the composition, which is from the TiB_2_ particles. We consider that there are some TiB_2_ particles attached to the surface of Al particles. O is present as the surface oxide of the Al particles.

The dense pores on the surface of the Al particles indicate that the dissolving reaction of the Al matrix in the HCl solution is a pitting reaction [35]. A pitting reaction often occurs on metals which have a non-uniform protective film on their surface. For example, when the surface of Al is oxidized, this passivation film should serve as a protective layer which prevents Al from reacting with HCl. In that way, HCl can only corrode Al in some areas where the passivation layer is damaged, causing the presence of etch pits. Since the sample is polished and the oxide film is mostly removed by strong acid before the dissolution experiment, etch pits are not caused by the oxide passivation layer but by a TiB_2_ adsorption layer.

The adsorption of TiB_2_ particles on the Al surface can be classified as either chemical adsorption or physical adsorption. Chemical adsorption mainly results from the in-situ synthesis process. After the generation of TiB_2_ particles in the Al melt, these TiB_2_ particles can be the nucleation site for Al crystals during its solidification, and a strong Al/TiB_2_ interface should be formed at the same time [36].

Physical adsorption mainly results from environmental stability in the later stage of the dissolution reaction. In the initial stage of the reaction, the HCl reacts with Al violently, releasing a large amount of heat. The increasing temperature accelerates the reaction rate, and also promotes the convection of the liquid. In this stage, most TiB_2_ particles are in dynamic motion. In the later stage of the reaction, with the decreasing HCl concentration and reaction area, the reaction rate decreases. TiB_2_ and Al particles are deposited at the beaker’s bottom, and the tiny TiB_2_ particles tend to adsorb on the surface of the Al particles. The adsorption of TiB_2_ on the Al particles prevents the Al from wholly reacting with the HCl, which can cause the formation of etch pits on the Al particles. Based on the above analysis, the core factor to increase the dissolution rate of the Al matrix is to prevent the adsorption of TiB_2_ particles on the Al surface. Since it is more difficult to carry out the desorption of chemical adsorption, the improvement of the dissolution process is mainly handled by facilitating the desorption of physical adsorption.

Next, alternate methods of normal ultrasonic treatment and magnetic stirring were adopted in the process of dissolving the Al matrix in HCl, which we expected to greatly accelerate the dissolution reaction. Generally, the 5 g TiB_2_/Al composite was entirely dissolved within 4 h. The alternated uses of normal ultrasonic treatment and magnetic stirring we considered to promote the completed reaction for the following reasons:

(1) Ultrasound can cause the formation, expansion, contraction, and implosion of micro gas nuclei in the liquid phase. This is also called the cavitation effect [37]. The cavitation effect of ultrasound can generate high temperature and pressure locally, which directly accelerates the rate of chemical reaction. Additionally, the formation of cavitation bubbles can promote the intensive mixing of solid and liquid, facilitating the desorption of TiB_2_ particles from the Al surface. Under such circumstances, a larger Al particle active surface area is exposed, and hence the reaction rate increases.
(2) Magnetic stirring mainly plays a role in promoting liquid convection. Under magnetic stirring, the dispersion of TiB_2_ particles in the liquid phase is a dynamic process, preventing them from achieving a stable physical adsorption process on the Al surface.

Compared with normal ultrasound treatment, magnetic stirring promotes the chemical reaction by a smaller degree. However, the prolonged use of the ultrasonic device should induce an increased temperature generated by the ultrasonic waves, thus reducing the service life of the device. Therefore, both methods are alternately used to accelerate the dissolution of the Al matrix.

#### 3.1.2. Drying Process Optimization

After the extraction process, an uncontrolled TiB_2_ suspension in deionized water can be obtained. Such a suspension should be dried to obtain dry TiB_2_ powder. The necessity of the drying process can be concluded thusly:

(1) The particle concentration of the uncontrolled suspension obtained from the extraction process is unknown, which presents difficulties to the subsequent optimization process of added surfactant concentration. In addition, NSA characterization requires a specific particle concentration range of suspension. Therefore, the drying process is necessary to obtain a certain particle concentration in the suspension.
(2) There is a large difference in particle size at different positions in the uncontrolled suspension. The poor uniformity in this suspension prevents the collection of samples which reach statistical significance for particle size characterization, and hence causes statistical error in our results. Indeed, the drying process can alleviate the randomness of each sample in the experiment, and, hence, the correlated NSA result should be more statistically significant.

During the drying process, the liquid surface tension can cause the interface to shrink, which increases the agglomeration tendency of TiB_2_ particles. Additionally, strong hydrogen bonds should form between water molecules on the TiB_2_ surface during the drying process, causing a bridging effect between the particles and leading to hard agglomeration among particles [38]. Therefore, the drying process should be properly controlled, and subsequently either vacuum-drying or freeze-drying treatments were performed on the extracted powder samples.

**Figure 3:**
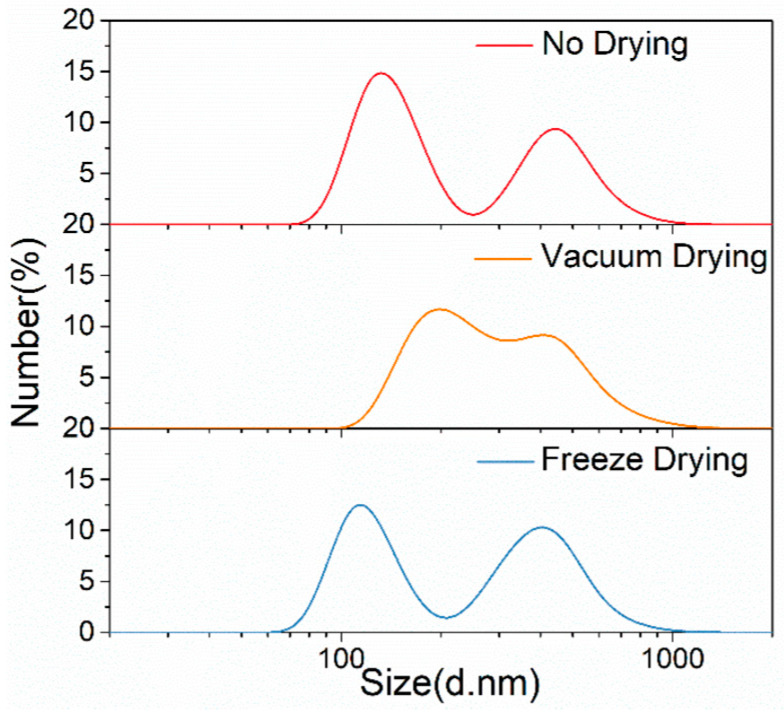
Size distributions of TiB_2_ particles from the undried, vacuum-dried, and freeze-dried samples by the NSA method.

Figure 3 and Table 1 present the size distributions of TiB_2_ particles from the undried, vacuum-dried and freeze-dried samples. In the undried sample, we conducted a direct size characterization without performing any drying process on the uncontrolled TiB_2_ suspension. It can be found that the sizes of three samples characterized by NSA all present an obvious bimodal distribution for TiB_2_ particles. For the TiB_2_ particles from the undried sample, the large-sized particle peak position was 441 nm and the small-sized peak was at 133 nm; its number-average diameter was 278 ± 14 nm. The formula for calculating the number-average diameter (*d*_number-average)_ for the TiB_2_ particles is shown in the following equation:

(1) $$d_{\mathrm{number}\text{-}\mathrm{average}}=\sum_{i=1}^{N}d_{i}/N,$$

where *d_i_* is the diameter of the *i*-th particle; *N* is the total number of particles.

For the vacuum-dried sample, the small-sized TiB_2_ particle peak position shows an obvious right (larger size) shift, and the size bimodal distribution is less prominent. Additionally, the number-average diameter of the TiB_2_ particles from the vacuum-dried sample was ~16% larger than that of the undried sample, indicating that the vacuum-drying process aggravates the clustering tendency of TiB_2_ particles.

For the freeze-dried sample, the positions of the small-sized and large-sized peaks for the TiB_2_ particles are slightly smaller than those of the undried sample. There is also a very small difference in the number-average diameter value for the TiB_2_ particles between the freeze-dried sample and undried sample. These results show that the freeze-drying process should not cause significant agglomeration of particles.

Temperature is the main factor leading to the above results. For the vacuum-drying process, the temperature is controlled at 60 °C. This higher temperature leads to higher surface activity, and, hence, a greater probability of bonding between particles. For the freeze-drying process, the temperature is held below 0 °C and the liquid water should turn into ice. The density difference between water and ice can cause an increase in volume, which directly leads to an increasing distance between particles, and, hence, a decrease in the agglomeration tendency. In addition, the agglomeration can be further suppressed at lower temperature. In summary, the freeze-drying process can greatly inhibit the agglomeration tendency of TiB_2_ particles, which can lay a foundation for further particle size characterization.

### 3.2. Chemical Dispersion Process Optimization

#### 3.2.1. Optimization of Surfactant Types and Suspension pH

Preliminarily, once the acquisition of the TiB_2_ powder by freeze-drying was achieved, a quantitative amount of TiB_2_ powder was added into deionized water, and normal ultrasound treatment (120 W) was used to obtain an initial TiB_2_ suspension (0.2 mg/mL, undispersed state). Furthermore, in order to achieve stable dispersion of the TiB_2_ particles, optimization of surfactant types and suspension pH values was performed thereafter.

**Figure 4:**
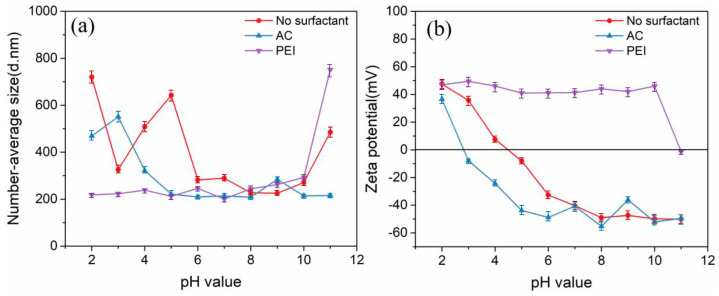
(**a**) Number-average diameters (NSA method) and (**b**) Zeta potentials of TiB_2_ particles on dependences of pH values and surfactant additions in related suspensions.

According to different charges of the hydrophilic groups, surfactants can be divided into anionic and cationic surfactants. In this study, both an anionic surfactant (ammonium citrate (CA (Sinopharm Group, China))) and cationic surfactant (polyethyleneimine (PEI (Sigma-Aldrich, Darmstadt, Germany))) were used to explore their dispersion effects on TiB_2_ particles under different suspension pH values. Figure 4 shows the number-average diameters (NSA method) and zeta potentials of TiB_2_ particles under different pH values and surfactant additions.

The characterized number-average diameters of TiB_2_ particles can indirectly reflect the dispersion state of TiB_2_ particles in the liquid phase. For example, the higher degree of particle dispersion in the suspension has corresponded to a smaller measured number-average diameter of the particle. Therefore, the number-average diameter values can reflect the dispersion degree of TiB_2_ in the suspension under different conditions (Figure 4a).

The pH value of the suspension plays an important role in the dispersion degree of TiB_2_ particles, which can be reflected as follows:

(1) For the initial TiB_2_ suspension (no surfactant), the number-average diameter of TiB_2_ is in the range of 200–300 nm with a suspension pH range of 6–10, which shows a good dispersion state of TiB_2_ in related suspensions.
(2) At pH values of 2/5/11, the number-average diameters of TiB_2_ increase sharply, which reflects serious agglomeration among TiB_2_ particles in the suspension.

The type of added ionic surfactant also has a significant effect on TiB_2_ dispersion under different pH conditions (Figure 4a). For example, with PEI addition, we find a number-average diameter for TiB_2_ in the range of 200–300 nm under a pH range of 2–10 in the TiB_2_ suspension. Comparably, AC addition can only have the same dispersion effect under the pH range of 5–8 in the TiB_2_ suspension. This means that PEI addition can maintain a good dispersion state of TiB_2_ particles over a larger pH range compared to AC addition into the TiB_2_ suspension.

The zeta potential can reflect the surface charging of particles and characterize the electrostatic repulsion effect among particles in the liquid phase [28,39]. A greater absolute value of the zeta potential means a larger amount of charge on the particles, leading to greater repulsion among particles. Hence, the zeta potential can explain the core reason for the dispersion effect induced by the surfactant. The zeta potentials of the TiB_2_ particles should depend on both surfactant additions and pH values in the suspensions, as shown in Figure 4b. Clearly, the isoelectric point of TiB_2_ without surfactant addition is 4.5, which means that TiB_2_ is negatively charged when the pH is greater than 4.5 and positively charged when the pH is less than 4.5 (Figure 4b). This feature explains the improved dispersion effect induced by PEI addition to the suspension. Since TiB_2_ is more likely to be negative charged in the liquid phase, the cationic surfactant PEI can more easily adsorb on the surface of TiB_2_ particles. Additionally, owing to the charge overcompensation effect of the polymer chain of PEI [40], the zeta potential of the TiB_2_ particles changes from negative to positive and maintains a high value over a wider pH range (Figure 4b).

**Figure 5:**
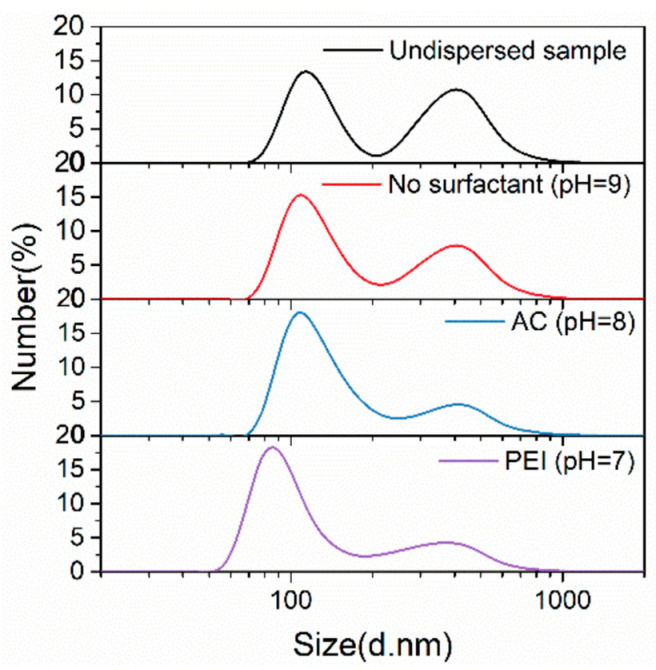
Size distributions of TiB_2_ particles (NSA method) under different surfactant additions with optimal pH values.

For the three surfactant-added suspensions (No surfactant/PEI/AC), the TiB_2_ size distribution at each optimal pH value is explored and compared with the undispersed sample, which is maintained without surfactant addition at pH = 7. The results are shown in Figure 5 and Table 2.

In the initial TiB_2_ suspension (no surfactant), adjusting the solution pH value from 7 to 9 can have a positive effect on the dispersion of TiB_2_ particles, with the measured number-average diameter of TiB_2_ dropping from 276 ± 13 nm to 242 ± 12 nm. Adding PEI surfactant (pH = 7) into the suspension, the dispersion state of TiB_2_ is optimal, in which the measured number-average diameter is reduced to 173 ± 9 nm, and the positions of the small-sized and large-sized peak move left to 85 nm and 373 nm, respectively.

From the above results and discussions, it can be determined that TiB_2_ particles are more likely to be negatively charged on the surface in an aqueous suspension. Therefore, the cationic surfactant PEI should be more easily adsorbed on the TiB_2_ surface, and, hence, be more effective than the anionic surfactant AC on the dispersion effect.

#### 3.2.2. Optimization of the Surfactant Molecular Weight and Concentration

**Figure 6:**
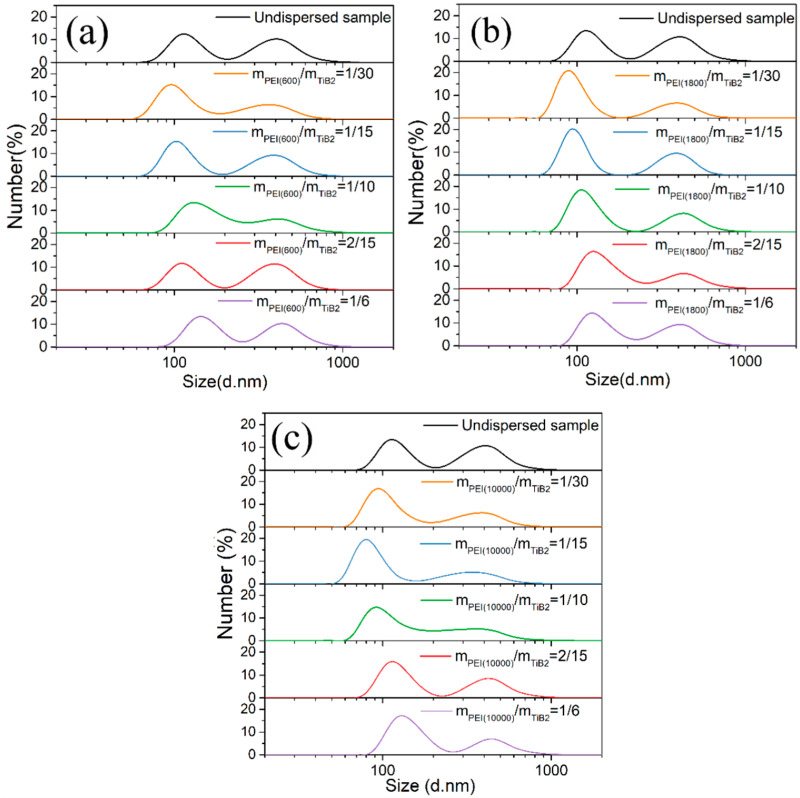
Size distributions of TiB_2_ particles (by NSA method) under different PEI concentrations with PEI molecular weights of (**a**) 600, (**b**) 1800 and (**c**) 10000.

Once the surfactant type and its corresponding optimal pH value are determined, it is necessary to choose the appropriate molecular weight and concentration of the chosen surfactant in the suspension. The specific effect of different molecular weights and concentrations of PEI on the dispersion degree of TiB_2_ in suspension can be reflected, as shown in Figure 6.

In Figure 6, it can be observed that with the increasing PEI concentrations of three molecular weights (M.W. 600/1800/10000) in the suspension, the measured size distribution curves of the TiB_2_ particles mostly shift to the left (smaller size), and then to the right (larger size). This phenomenon means that dispersion effect of PEI on TiB_2_ particles first increases, and then decreases with an increased PEI concentration in the suspension.

The main reason for the above phenomenon can be concluded as follows:

(1) When the PEI concentration is low in the suspension, the amount of adsorbed PEI on the TiB_2_ surface should be larger as the PEI concentration increases. In that case, the increased charge on the TiB_2_ should result in a greater electrostatic repulsion between particles, and, hence, improve the dispersion degree of TiB_2_ particles.
(2) When the PEI concentration is too high in the suspension, owing to the entanglement between the polymer chains attached to the TiB_2_ particles, the tendency to agglomerate among particles should increase [41].

**Figure 7:**
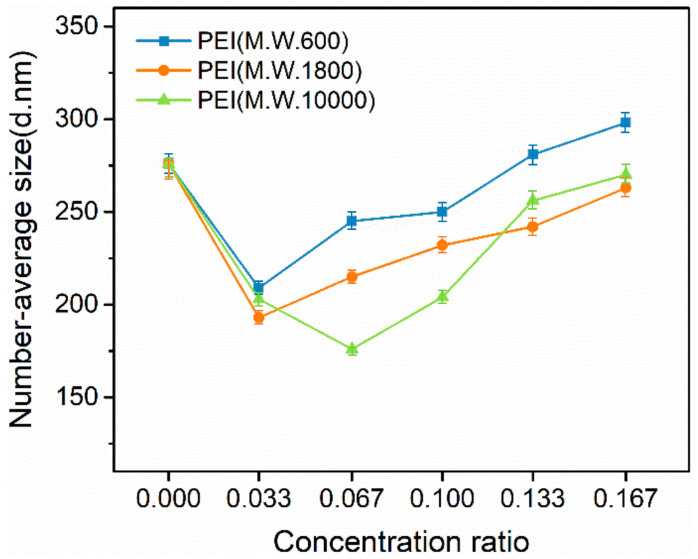
Number-average diameters of TiB_2_ particles (NSA method) in the added PEI (M.W. 600/1800/10000) suspensions.

Figure 7 shows the change in the measured number-average diameters of TiB_2_ particles (NSA method) under different concentrations and different molecular weights of PEI. From these results, both PEI (M.W. 600) and PEI (M.W. 1800) achieve the optimal effect at the concentration ratio of m_PEI_/m_TiB2_ = 1/30 in the suspension, and PEI (M.W. 10000) achieves the optimal effect at the concentration ratio of m_PEI_/m_TiB2_ = 1/15 in the suspension. The optimal concentration of PEI (M.W. 10000) is higher than those of PEI (M.W. 600) and PEI (M.W. 1800). This is because, under the same concentration conditions, the total amount of PEI with the larger molecular weight is less than that of the PEI with the other two smaller molecular weights. In this case, a higher concentration of PEI (M.W. 10000) should be used to reach the saturated adsorption state on TiB_2_ particles in the suspension.

In Table 3, PEI (M.W. 10000) added at a m_PEI_/m_TiB2_ = 1/15 ratio in the suspension can make TiB_2_ particles achieve a superior dispersion degree. In comparison with the initial TiB_2_ suspension (undispersed state), the measured number-average diameter of the TiB_2_ particles decreases from 276 ± 13 nm to 176 ± 9 nm, with the positions of the small-sized and large-sized peaks shifting left to 80 nm and 348 nm, respectively.

The phenomenon that the dispersion effect of the PEI (M.W. 10000) is better than the PEI (M.W. 600) and PEI (M.W. 1800) in the suspension can be explained in relation to the following aspects:

(1) For polymers, a longer polymer chain means more charged groups in a molecule. Therefore, under the same saturated adsorption state, the surfactant with the larger molecular weight can provide more charge to the particles. Hence, the electrostatic repulsion between particles becomes greater [39]. This can also be reflected by the zeta potentials of the TiB_2_ particles in Table 3.
(2) As the molecular weight increases, the polymer chain should be longer, which makes the charged layer of TiB_2_ particles thicker and provides greater steric hindrance in the dispersion of particles [42].

### 3.3. Physical Dispersion Process Optimization

**Figure 8:**
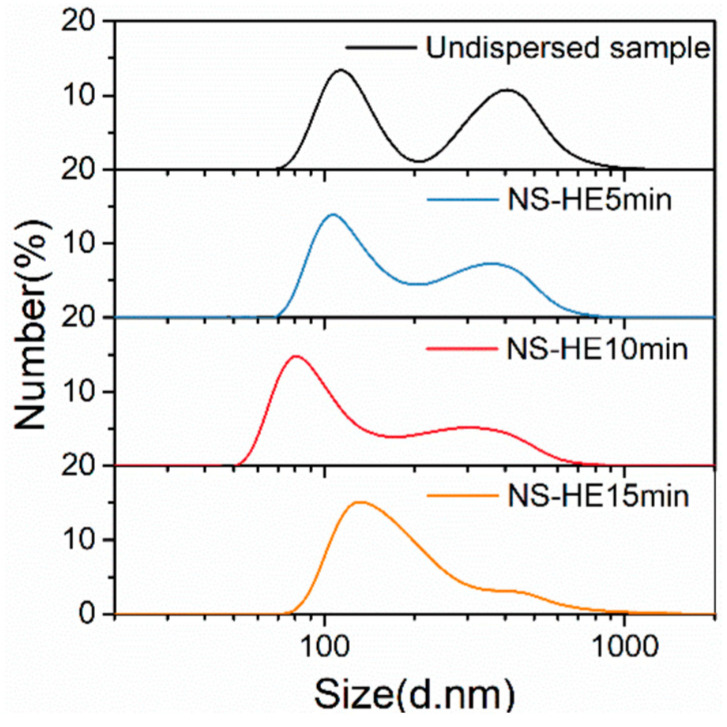
Size distributions of TiB_2_ particles (NSA method) under high-energy ultrasound (HE) of different durations and with no surfactant (NS) in the suspensions.

Surfactant addition can largely inhibit the agglomeration of particles in the liquid phase. However, for hard agglomerations formed during the synthesis and drying processes, they are difficult to disperse only by chemical dispersion. High-energy ultrasound, which can produce cavitation, acoustic flow, local high temperature, and pressure, is expected to break the hard agglomerations and further improve the dispersion degree of TiB_2_ particles in the suspension [37]. The measured size distributions of TiB_2_ particles after high-energy ultrasonic treatment for different durations are shown in Figure 8 and Table 4.

The application of high-energy ultrasound for three different durations (5/10/15 min) all have a considerable dispersion effect on TiB_2_ suspension. In addition, it can be observed that, with the increase in duration of high-energy ultrasound application, the measured size distribution curve first shifts to the left (smaller size) and then to the right (larger size), with the corresponding dispersion degree of TiB_2_ particles first increasing and then decreasing. The reasons for this phenomenon are as follows:

(1) At the beginning, as the duration of high-energy ultrasound application increases, more and more particle agglomerations can be dispersed, and the dispersion effect of the particles is improved.
(2) As the duration of high-energy ultrasound further increases, the system energy increases and the temperature rises, which leads to more intense Brownian movement of the particles. Herein, the possibility of collision between particles and formation of agglomerations increases instead, which alleviates the dispersion degree of particles.

It also can be seen that the optimal dispersion degree of TiB_2_ particles is achieved by 10 min of high-energy ultrasonic treatment. In this case, the measured number-average diameter of the TiB_2_ particles is 178 ± 9 nm, which is reduced by 35.5% as compared to the initial TiB_2_ suspension (Undispersed state).

### 3.4. Chemical–Physical Collaborative Dispersion Process Optimization

**Figure 9:**
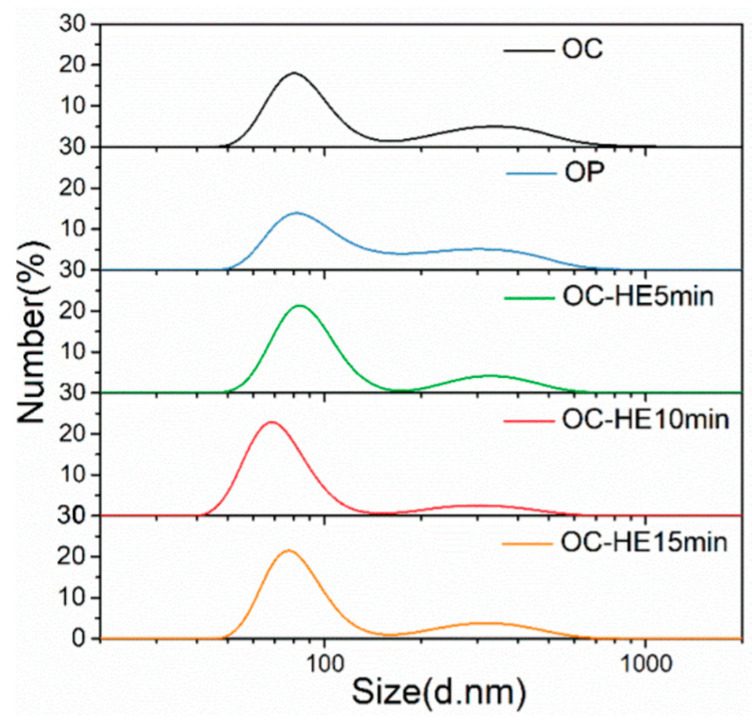
Size distributions of TiB_2_ particles (by NSA method) under optimal chemical dispersion (OC), optimal physical dispersion (OP), and optimal chemical dispersion and high-energy ultrasound (OC–HE) of different durations.

On account of the chemical–physical collaborative dispersion process, our study explored the dispersion effect of different high-energy ultrasound durations on the TiB_2_ particles, based on the optimal chemical dispersion condition (pH = 7, adding PEI (M.W. 10000) with m_PEI_/m_TiB2_ = 1/15). Figure 9 shows the size distributions of TiB_2_ particles (NSA method) under optimal chemical dispersion (OC), optimal physical dispersion (OP), and optimal chemical dispersion and high-energy ultrasound (OC–HE) of different durations.

Based on the OC dispersion conditions, applying 10 min of high-energy ultrasound can allow the TiB_2_ particles reach the maximum dispersion degree. Under this condition (OC–HE 10 min), the measured number-average diameter is 111 ± 6 nm, which is reduced by 59.8% as compared to the initial TiB_2_ suspension (Undispersed state). The positions of the small-sized and big-sized peaks for the TiB_2_ particles are 68 nm and 292 nm, respectively.

Comparing the measured size distributions of TiB_2_ particles under various conditions in Figure 9 and Table 5, it can be concluded that under the chemical–physical collaborative dispersion conditions, the dispersibility of TiB_2_ particles is significantly improved over either chemical-only dispersion or physical-only dispersion states. In other words, in the process of dispersing TiB_2_ particles in the liquid phase, a combination of chemical and physical dispersion is necessary. For physical dispersion, the use of high-energy ultrasound can open the soft and hard agglomerations. For chemical dispersion, adding a surfactant and adjusting the pH of the solution should further provide electrostatic hindrance and steric hindrance to maintain the stable dispersion of TiB_2_ particles. Finally, the controlled processes involved with either physical or chemical dispersions have shown excellent measurement reproducibility, based on their clear dispersion mechanisms.

### 3.5. Comparison of Different Particle Size Measurements

**Figure 10:**
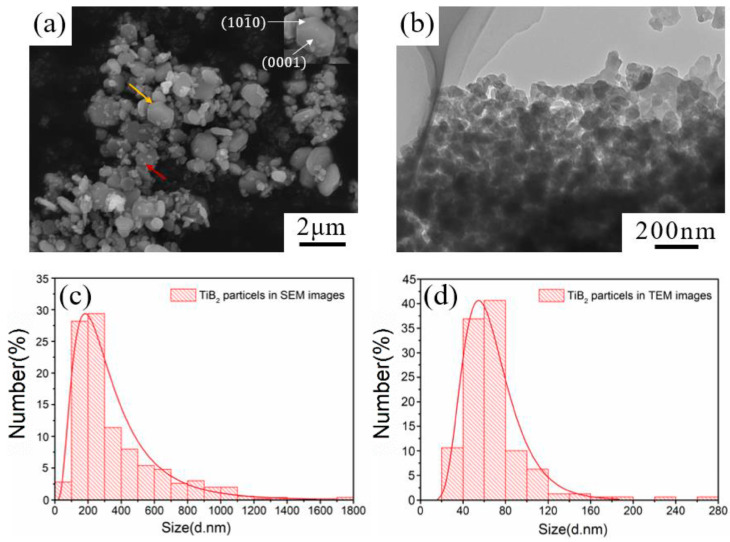
(**a**) SEM image, (**b**) TEM image, the size distributions characterized by (**c**) SEM and (**d**) TEM of TiB_2_ particles.

From the above results, the optimal dispersion state of TiB_2_ can be achieved by combining chemical and physical dispersion methods. In this case, the size distribution of TiB_2_ particles characterized by NSA is bimodal, with the small and large size peaks located at 68 nm and 292 nm, respectively. The measured number-average diameter is 111 ± 6 nm, which is reduced by 59.8% compared to the initial TiB_2_ suspension (undispersed state). In order to verify the effectiveness of the dispersion methods and the accuracy of the size results characterized by NSA, both SEM and TEM have been further used to obtain size information for the TiB_2_ particles. The corresponding results are shown in Figure 10.

In the SEM image of TiB_2_ particles (Figure 10a), small-sized TiB_2_ particles show nearly spherical morphology (indicated by the red arrow) and large-sized TiB_2_ particles exhibit an obvious hexagonal plate structure (indicated by the yellow arrow). The difference in particle morphologies is mainly due to the different growth behaviors of the crystal. When the TiB_2_ initially nucleates in the melt, since the growth conditions are similar to each other, there are rarely obvious growth differences in different crystal plane, and the particles are nearly spherical. As the crystal grows, the crystal tends to grow rapidly along a close-packed direction [11$\bar{2}$0]. Correspondingly, the close-packed surface (0001) of TiB_2_ is gradually exposed to the outside, which leads to characteristic hexagonal flakes [43]. From Figure 10a,c, it can be observed that the size distribution of TiB_2_ particles is very wide (0–1800 nm), and most of the small-sized particles are attached to the surface of the large-sized particles. In addition, the peak of the log-normal distribution fitting result from SEM is around 185 ± 9 nm.

In the TEM image of TiB_2_ particles (Figure 10b), most of the particles are below 100 nm, and it is difficult to find large-sized (>200 nm) particles in view. From the size information characterized by TEM (Figure 10d), the size distribution of TiB_2_ particles is between 20–280 nm, which is relatively narrow. The log-normal distribution fitting peak is located around 55 ± 3 nm.

Combining all the results obtained by the three size-measuring methods, it can be found that the size distributions of in-situ TiB_2_ particles characterized by SEM and TEM are unimodal, while the TiB_2_ distribution characterized by NSA is bimodal. In addition, the small-sized peak characterized by NSA matches the result obtained by TEM, while the large-sized peak characterized by NSA matches the result obtained by SEM. This phenomenon shows that, under good dispersion conditions, NSA can coordinate with the wide size distribution of in-situ TiB_2_ particles, and is well-applied to the size characterization of TiB_2_.

However, the position of the small-sized peak measured by NSA (68 nm) is slightly larger than that of the TEM result (55 ± 3 nm), and the position of the large-sized peak (292 nm) is larger than that of the SEM result (185 ± 9 nm). The reasons for these discrepancies are considered as following:

(1) The result obtained from NSA is the hydrodynamic size of the particles, which is generally lightly larger than the actual size of the particles [44].
(2) After the dispersion process, TiB_2_ particles may be well-dispersed but not completely dispersed in the suspension. Some of the larger-sized particle information may come from some undispersed agglomerations, which also causes the peak positions to be larger than those obtained by SEM and TEM. Therefore, further research should be performed to find a more effective cationic surfactant for chemical dispersion, or a novel treatment for the physical dispersion of TiB_2_ suspensions.

## 4. Conclusions

In order to use NSA to obtain the precise size distribution of in-situ TiB_2_ particles, controlled size characterization processes (i.e., the extraction/drying process of TiB_2_ particles, and dispersion treatment on the TiB_2_ suspension) are explored, in which the dispersion techniques and mechanisms for TiB_2_ suspension are focused on. The main conclusions are drawn as following:

(1) In the extraction process, the alternated applications of magnetic stirring and normal ultrasound treatment were proved to accelerate the dissolution of the Al matrix in HCl solution. Furthermore, freeze-drying was found to minimize the tendency of agglomeration among TiB_2_ particles during the drying process.
(2) For chemical dispersion, a specified TiB_2_ suspension (pH = 7/PEI (M.W. 10000) with m_PEI_/m_TiB2_ = 1/15) can provide TiB_2_ particles with maximum electrostatic and spatial steric hindrance. The corresponding number-average diameter of TiB_2_ characterized by NSA is 176 ± 9 nm, which is reduced by 36.2% compared to the initial TiB_2_ suspension (Undispersed state).
(3) For physical dispersion, the best duration of high-energy ultrasound application is found to be 10 min. The corresponding number-average diameter of TiB_2_ is 178 ± 9 nm, which is reduced by 35.5% compared to the initial TiB_2_ suspension (Undispersed state).
(4) For combined chemical and physical dispersion methods, the optimal dispersion state of TiB_2_ particles can be achieved in the liquid phase. In this case, the number-average diameter of TiB_2_ characterized by NSA is 111 ± 6 nm, which is reduced by 59.8% compared to the initial TiB_2_ suspension (Undispersed state).
(5) Under the optimal dispersion condition, the bimodal size distribution of TiB_2_ was characterized by NSA for the first time. In such a bimodal distribution, the small-sized and large-sized peaks of TiB_2_ are located at 68 nm and 292 nm, which can be matched to the results characterized by TEM and SEM, respectively.

Generally, this work shows that, under the precondition of achieving good dispersion of TiB_2_ particles, NSA can address the characteristics of the wide size distribution of TiB_2_ particles and obtain more accurate size information than other available methods. 

## Figures and Tables

**Table 1:** Detailed size information of TiB_2_ particles from the undried, vacuum-dried, and freeze-dried samples by the NSA method.

	Position of Small-Size Peak (nm)	Position of Large-Size Peak (nm)	*d*_number-average_ (nm)
No drying	133	441	278 ± 14
Vacuum-drying	193	423	323 ± 16
Freeze-drying	114	407	276 ± 13

**Table 2:** Detailed size information of TiB_2_ particles (NSA method) under different surfactant additions with optimal pH values.

	Position of Small-Size Peak (nm)	Position of Large-Size Peak (nm)	*d*_number-average_ (nm)
Undispersed sample	114	407	276 ± 13
No surfactant (pH = 9)	109	405	242 ± 12
AC (pH = 8)	108	407	198 ± 10
PEI (pH = 7)	85	373	173 ± 9

**Table 3:** Detailed size information (NSA method) and zeta potentials of TiB2 particles under different concentrations of added PEI at corresponding optimal concentrations.

	Optimal Concentration Ratio (m_PEI_/m_TiB2_)	Position of Small-Size Peak (nm)	Position of Large-Size Peak (nm)	*d*_number-average_ (nm)	Zeta Potential (mv)
Undispersed sample	/	114	407	276 ± 13	−41
PEI (M.W. 600)	0.033	94	378	209 ± 10	+37
PEI (M.W. 1800)	0.033	89	381	193 ± 10	+45
PEI (M.W. 10000)	0.067	80	348	176 ± 9	+54

**Table 4:** Detailed size information for TiB2 particles (NSA method) under high-energy ultrasound (HE) of different durations and no surfactant (NS) in the suspensions.

	Position of Small-Size Peak (nm)	Position of Large-Size Peak (nm)	*d*_number-average_ (nm)
Undispersed sample	114	407	276 ± 13
NS-HE 5 min	109	342	227 ± 11
NS-HE 10 min	78	295	178 ± 9
NS-HE 15 min	141	458	214 ± 11

**Table 5:** Detailed size information of TiB_2_ particles (NSA method) under optimal chemical dispersion (OC), optimal physical dispersion (OP), and optimal chemical dispersion and high-energy ultrasound (OC–HE) of different durations.

	Position of Small-Size Peak (nm)	Position of Large-Size Peak (nm)	*d*_number-average_ (nm)
OC	80	348	176 ± 9
OP	78	295	178 ± 9
OC–HE 5 min	82	316	141 ± 7
OC–HE 10 min	68	292	111 ± 6
OC–HE 15 min	77	310	140 ± 7

## Data Availability

The data are contained within the article.
